# In-plane anisotropy of graphene by strong interlayer interactions with van der Waals epitaxially grown MoO_3_

**DOI:** 10.1126/sciadv.adg6696

**Published:** 2023-06-07

**Authors:** Hangyel Kim, Jong Hun Kim, Jungcheol Kim, Jejune Park, Kwanghee Park, Ji-Hwan Baek, June-Chul Shin, Hyeongseok Lee, Jangyup Son, Sunmin Ryu, Young-Woo Son, Hyeonsik Cheong, Gwan-Hyoung Lee

**Affiliations:** ^1^ Department of Materials Science and Engineering, Seoul National University, Seoul 08826, South Korea.; ^2^ Department of Physics, Inha University, Incheon 22212, South Korea.; ^3^ Department of Physics, Sogang University, Seoul 04107, South Korea.; ^4^ School of Computational Sciences, Korea Institute for Advanced Study, Seoul 02455, South Korea.; ^5^ Department of Chemistry, Pohang University of Science and Technology, Pohang 37673, South Korea.; ^6^ Korea Research Institute of Standards and Science, Daejeon 34113, South Korea.; ^7^ Functional Composite Materials Research Center, Korea Institute of Science and Technology (KIST), Jeonbuk 55324, South Korea.; ^8^ Division of Nano and Information Technology, KIST School University of Science and Technology (UST), Jeonbuk 55324, South Korea.

## Abstract

van der Waals (vdW) epitaxy can be used to grow epilayers with different symmetries on graphene, thereby imparting unprecedented properties in graphene owing to formation of anisotropic superlattices and strong interlayer interactions. Here, we report in-plane anisotropy in graphene by vdW epitaxially grown molybdenum trioxide layers with an elongated superlattice. The grown molybdenum trioxide layers led to high p-doping of the underlying graphene up to *p* = 1.94 × 10^13^ cm^−2^ regardless of the thickness of molybdenum trioxide, maintaining a high carrier mobility of 8155 cm^2^ V^−1^ s^−1^. Molybdenum trioxide–induced compressive strain in graphene increased up to −0.6% with increasing molybdenum trioxide thickness. The asymmetrical band distortion of molybdenum trioxide–deposited graphene at the Fermi level led to in-plane electrical anisotropy with a high conductance ratio of 1.43 owing to the strong interlayer interaction of molybdenum trioxide–graphene. Our study presents a symmetry engineering method to induce anisotropy in symmetric two-dimensional (2D) materials via the formation of asymmetric superlattices with epitaxially grown 2D layers.

## Teaser

Epitaxially grown MoO_3_ on graphene induces in-plane conductance anisotropy as well as high strain, doping, and carrier mobility.

## INTRODUCTION

van der Waals (vdW) epitaxy is a widely used technique for fabricating two-dimensional (2D) epitaxial layers on a variety of growth templates with crystallographic alignment (*1*–*3*). The major advantages of vdW epitaxy originate from the dangling bond–free interface and relatively weak vdW interactions between the epilayers and growth templates (*4*), enabling the growth of epilayers with a remarkable mismatch of lattice (*5*) and symmetry (*6*–*8*) without substantial interfacial strain or misfit dislocations. The interlayer interaction is of particular importance in vdW epitaxy with symmetry mismatch due to the formation of anisotropic superlattices (*9*–*12*) and directional strain during a cooling procedure (*13*).

Here, we report the in-plane anisotropy in graphene induced by the strong interlayer interaction with vdW epitaxially grown α-MoO_3_. The strong interlayer interaction at the vdW interface helped modulate the mechanical and electrical properties of monolayer graphene. The epilayer and graphene template with different thermal expansion coefficients (TECs) exerted a high compressive strain on graphene, which increased up to −0.6% with increasing MoO_3_ thickness. Meanwhile, the graphene was highly p-doped (*p* = 1.94 × 10^13^ cm^−2^) by the charge transfer from MoO_3_ regardless of the MoO_3_ thickness. Notably, MoO_3_-deposited graphene exhibited in-plane anisotropy (conductance ratio of 1.43) in the electrical conductance owing to the crystal orientation–related periodic potentials by MoO_3_, maintaining a high carrier mobility of 8155 cm^2^ V^−1^ s^−1^. Our study shows that vdW epitaxial interface can induce the strong interlayer interaction and in-plane anisotropy, which can be used to modulate the crystal orientation–dependent properties of 2D materials.

## RESULTS

### Crystal structure of MoO_3_/graphene heterostructure

**Fig. 1.**
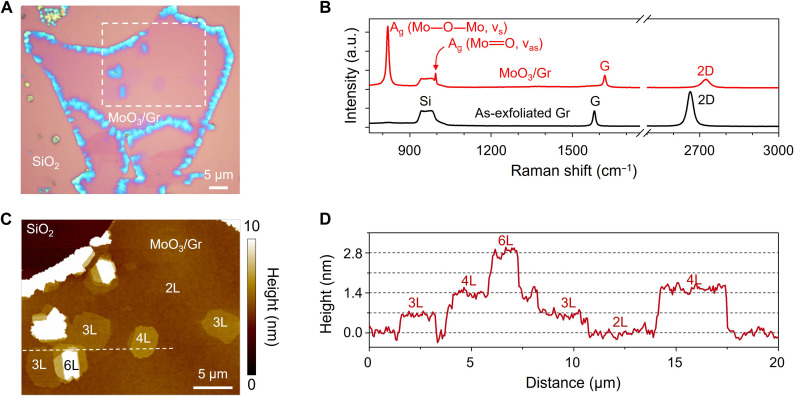
vdW epitaxial growth of MoO_3_ on monolayer graphene. (**A**) Optical microscope image of MoO_3_/Gr. (**B**) Raman spectra of as-exfoliated Gr (black) and MoO_3_/Gr (red). The notable features after MoO_3_ growth are the emergence of MoO_3_ Raman peaks at ~820 and ~990 cm^−1^, blueshift in the G and 2D peaks, and absence of D peak. a.u., arbitrary units. (**C**) AFM image of MoO_3_/Gr of the dashed area in (A). (**D**) AFM height profile along the white dashed line in (C). The heights of MoO_3_ islands correspond to multiple layers of MoO_3_ (~0.7 nm)

To investigate the interlayer interaction at a symmetry-mismatched epitaxial interface, we used orthorhombic α-MoO_3_ (*Pnma* space group) with in-plane anisotropic mechanical, electrical, and optical properties (*14*–*18*). MoO_3_ layers comprising MoO_6_ octahedral double layers (*a* = 3.96 Å and *c* = 3.70 Å) were stacked along the *b* axis with weak vdW forces (*14*, *19*–*22*). MoO_3_ was grown on exfoliated graphene by the thermal evaporation of the Mo film in the air (see Materials and Methods) (*23*). Figure 1A shows MoO_3_-grown monolayer graphene (MoO_3_/Gr). Figure 1B shows the Raman spectra of the as-exfoliated graphene and MoO_3_/Gr samples. Two main Raman peaks of MoO_3_ can be observed at ~817 and ~ 995 cm^−1^, corresponding to the stretching modes of doubly coordinated oxygen along the *a* axis (*24*, *25*). The absence of D peak, an indicator of defects in graphene, implies that there was no recognizable damage in graphene during MoO_3_ growth (*26*, *27*). However, G and 2D peaks, which are sensitive to doping and strain, shifted markedly after the deposition of MoO_3_. This indicates that the deposited MoO_3_ induced doping and strain in the underlying graphene. This is discussed later in the paper. The morphology and thickness of MoO_3_ were measured using atomic force microscopy (AFM), as shown in Fig. 1 (C and D). The graphene was fully covered by bilayer MoO_3_ (2L-MoO_3_) with a few thick islands, as reported previously (fig. S1) (*23*, *28*). The MoO_3_ surface was clean and flat without contamination or damage. The height profile in Fig. 1D (from the white dashed line in Fig. 1C) shows that the thickness of the monolayer MoO_3_ corresponds to half of the unit cell (~0.7 nm) (*14*, *19*–*22*).

### Epitaxial relationship between MoO_3_ and graphene

**Fig. 2.**
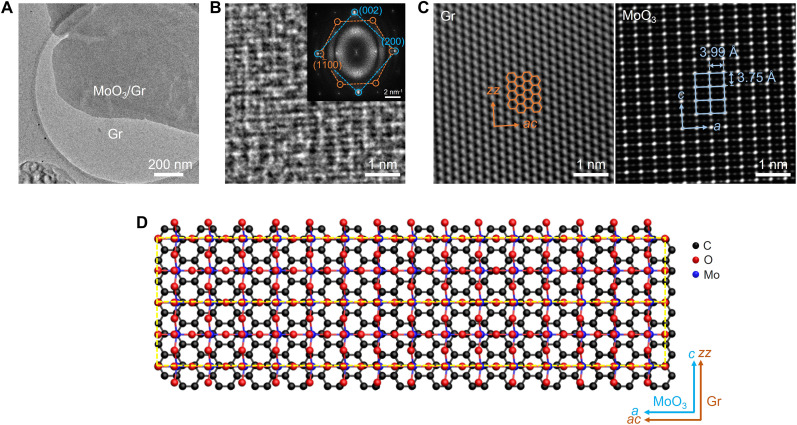
Epitaxial relationship between MoO_3_ epilayers and monolayer graphene growth template. (**A**) Low-magnification TEM image of MoO_3_/Gr. (**B**) High-resolution TEM image of MoO_3_/Gr. The inset shows the corresponding FFT image. Only a single set of rhombus pattern indicates the growth of single-crystal MoO_3_ (blue). In addition, the perfect alignment of MoO_3_ and graphene (orange) patterns demonstrates the epitaxial growth of MoO_3_ on monolayer graphene. (**C**) FFT-filtered images of graphene (left) and MoO_3_ (right). (**D**) Schematic of MoO_3_/Gr superlattices (yellow dashed boxes) based on their epitaxial relationship. The periodicity along the horizontal direction (*a* axis of MoO_3_ and *ac* direction of graphene) is approximately eight times greater than that along the vertical direction (*c* axis of MoO_3_ and *zz* direction of graphene).

To reveal the crystal structures and epitaxial relationship between MoO_3_ and Gr, we used spherical aberration-corrected transmission electron microscopy (Cs-TEM). The TEM image in Fig. 2A shows MoO_3_/Gr transferred onto a TEM grid with holes, where 2L-MoO_3_ is partially grown on the graphene. The high-resolution TEM image in Fig. 2B confirms the epitaxial growth of crystalline MoO_3_ on the graphene. The diffraction pattern obtained by fast Fourier transform (FFT), shown in the inset of Fig. 2B, verifies that the rhombic pattern (blue dashed line) of MoO_3_ is aligned with the hexagonal pattern (orange dashed line) of graphene. The (200) plane of MoO_3_ is parallel to the $\left(1\bar{1}00\right)$ plane of graphene (*29*, *30*). The FFT-filtered TEM images of graphene (left) and MoO_3_ (right) in Fig. 2C, obtained from each diffraction pattern, clearly show the growth of rectangular MoO_3_ unit cells with lattice parameters of 3.97 and 3.75 Å on the graphene with an epitaxial relationship: *a* and *c* axes of MoO_3_ are parallel to the armchair (*ac*) and zigzag (*zz*) directions of graphene, respectively. The atomic model in Fig. 2D shows the formation of a superlattice (indicated by yellow dashed lines) in the MoO_3_/Gr heterostructure with an epitaxial relationship. The long superlattice unit cell in MoO_3_/Gr can induce distinct periodicities along two directions of *ac* and *zz* in graphene (*9*–*12*).

### Modulation of doping and strain in graphene by MoO_3_ epilayer

**Fig. 3.**
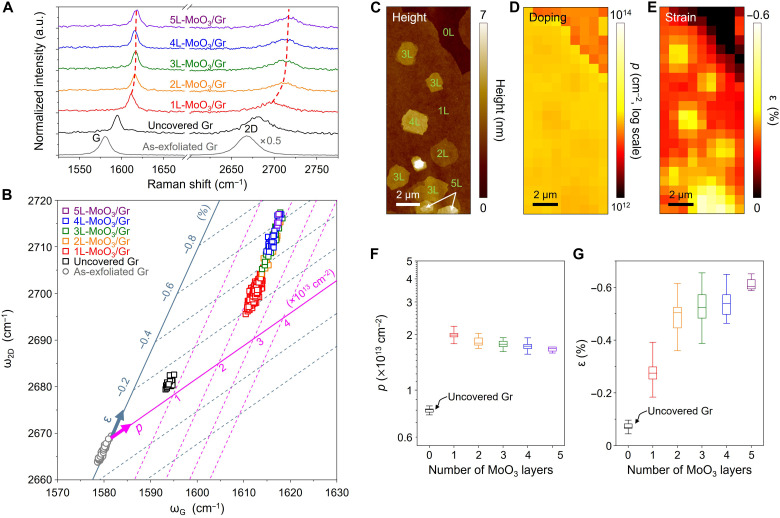
Hole concentration and strain in the MoO_3_-grown graphene. (**A**) Raman spectra of the as-exfoliated Gr (gray), uncovered Gr (black), and MoO_3_/Gr with various MoO_3_ thicknesses (rainbow colored, red to purple with increasing MoO_3_ thickness). For MoO_3_/Gr, the apexes of the G and 2D peaks are connected by dashed lines for visual guidance. (**B**) Correlation plot of the ω_2D_ − ω_G_ for the samples shown in (A). (**C**) AFM image of MoO_3_/Gr with various MoO_3_ thicknesses. (**D** and **E**) Mapping images of doping and strain of MoO_3_/Gr, respectively. The strain shows distinctive difference depending on the MoO_3_ thickness, whereas doping exhibits a difference depending only on the presence of MoO_3_ epilayers. (**F** and **G**) Plots of hole concentration and strain as a function of the number of MoO_3_ layers.

To investigate the interlayer interaction between MoO_3_ and graphene, we measured the Raman spectra of the graphene regions covered by MoO_3_ islands of different thicknesses (1L to 5L), as shown in Fig. 3A. For comparison, the Raman spectra of the as-exfoliated graphene (gray) and uncovered graphene (black) are also shown in Fig. 3A. The uncovered graphene is the region with no deposition of MoO_3_ after epitaxial growth. Although the uncovered graphene had no MoO_3_, there was a blue shift in the G and 2D peaks owing to the annealing effect (*31*). Notably, the MoO_3_/Gr region showed a marked blue shift in the G and 2D peaks compared to the as-exfoliated and uncovered graphene. Furthermore, the two peaks substantially blue-shifted with increasing MoO_3_ thickness. The marked shifts in the Raman peaks indicate a strong interlayer interaction between the MoO_3_ epilayers and graphene, leading to considerable doping and strain in graphene depending on the MoO_3_ thickness (*26*, *27*).

To separately measure the doping concentration (*p*) and strain (ε) of MoO_3_/Gr, we marked the positions of the G (ω_G_) and 2D (ω_2D_) peaks in the correlation plot of Fig. 3B. The doping and strain of graphene can be quantified by projecting ω_G_ and ω_2D_ to the doping concentration (magenta) and strain axes (gray), respectively (fig. S2) (*32*). As shown in Fig. 3B, the uncovered graphene is p-doped (*p* = 7.8 × 10^12^ cm^−2^) with a small compressive strain of −0.07% after the MoO_3_ growth process, which is due to the conformal contact of graphene on the SiO_2_ substrate by annealing under ambient condition (*31*, *33*, *34*). In the case of MoO_3_/Gr, the graphene is highly p-doped (*p* = 1.94 × 10^13^ cm^−2^) regardless of the MoO_3_ thickness, while the compressive strain increases with the number of MoO_3_ layers. Different MoO_3_ thickness dependence of the doping concentration and strain can be clearly visualized in Fig. 3 (C to E). Although MoO_3_ regions of different thicknesses were deposited on the graphene, as shown in the AFM image of Fig. 3C, the p-doping concentration of the graphene measured by the Raman peaks was maintained irrespective of the number of MoO_3_ layers, as shown in Fig. 3D. Meanwhile, thicker MoO_3_ epilayers resulted in a higher compressive strain, as shown in Fig. 3E.

To clearly show the MoO_3_ thickness dependence of doping and strain in graphene, we plotted the doping concentration and compressive strain as a function of the MoO_3_ thickness in Fig. 3 (F and G), respectively. The p-doping concentration of 1L-MoO_3_/Gr was almost twice higher than that of the uncovered graphene and almost invariant for 1L-MoO_3_ to 5L-MoO_3_ (Fig. 3F). This indicates that a high p-doping concentration of graphene can be achieved using only monolayer MoO_3_. This is attributed to the efficient charge transfer between MoO_3_ and graphene by the large and thickness-insensitive work function of MoO_3_ (*28*, *35*–*37*). In contrast, the compressive strain of MoO_3_/Gr clearly shows a dependency on the MoO_3_ thickness (Fig. 3G). The compressive strain exerted on graphene by MoO_3_ is due to the difference in the TECs between MoO_3_ and graphene (*14*, *38*, *39*) as well as the high lateral stiffness and friction of MoO_3_ (*28*, *40*, *41*). The strain of graphene increased, and the increment gradually decreased with the number of MoO_3_ layers. This may be due to the formation of a more rigid MoO_3_ structure with increasing thickness. Both the doping and strain of graphene induced by the MoO_3_ epilayer indicate a strong interlayer interaction between MoO_3_ and graphene with an epitaxial relationship.

### In-plane anisotropy of MoO_3_/Gr

**Fig. 4.**
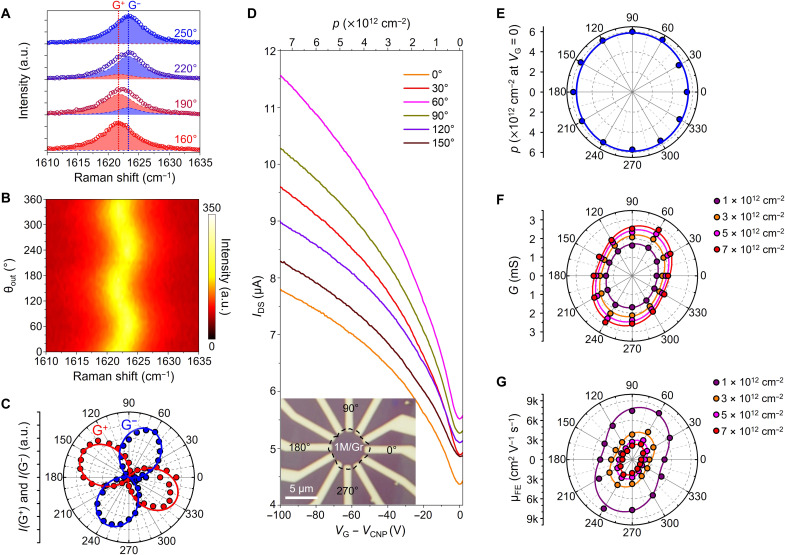
Anisotropy in graphene induced by MoO_3_ epilayers. (**A**) Fitting results of four representative angle-resolved polarized Raman spectra. The measured data are represented by the hollow circles, while the red and blue shaded areas correspond to the fitted results of the G^+^ and G^−^ peaks, respectively. The peak positions of G^+^ and G^−^ are indicated by the red and blue dotted lines, respectively. (**B**) Contour plot of the angle-resolved polarized Raman spectra of G peak as function of analyzer angle (θ_out_). (**C**) Intensities of G^+^ (red) and G^−^ (blue) peaks plotted in the polar coordinate. Spheres correspond to the intensities, and solid lines correspond to the results fitted to the theoretical expectation (*42*, *43*). (**D**) Orientation-dependent transfer characteristics of MoO_3_/Gr electric device. The inset shows an optical image of the MoO_3_/Gr electric device. (**E** to **G**) Polar coordinate plots of the hole concentration at zero-gate voltage (E), conductance (F), and field-effect mobility (G) of the MoO_3_/Gr device in (D), respectively. Conductance and field-effect mobility are measured under various carrier concentrations, which are represented with different colors.

The formation of an elongated superlattice and strong interlayer interaction at the MoO_3_-Gr heterointerface can induce crystal orientation–dependent modulation of the properties of graphene. To verify the orientation-dependent structural modulation, we measured the angle-resolved polarized Raman spectra of MoO_3_/Gr by rotating an analyzer (θ_out_) at a fixed polarizer (θ_in_) (see Materials and Methods for detailed information on angle-resolved polarized Raman spectroscopy). Figure 4A shows the G peaks of 3L-MoO_3_/Gr measured at four specific angles. The G peaks were deconvoluted into two peaks of G^+^ (red area) at 1621.7 cm^−1^ and G^−^ (blue area) at 1623.2 cm^−1^, which have eigenvectors parallel and orthogonal to the strain direction, respectively (fig. S3) (*42*–*44*). The intensities of these peaks vary as a function of θ_out_, resulting in a periodic shift in the convoluted G peak, as shown in the contour plot in Fig. 4B. The peak intensities of G^+^ (red circles) and G^−^ (blue circles) are plotted in polar coordinates in Fig. 4C. They show well-defined cos^2^ θ_out_ patterns that repel each other in accordance with theoretical predictions (solid lines in Fig. 4C), indicating the presence of a uniaxial strain in graphene (*42*–*44*). Therefore, our observations from the polarized Raman spectra show that the vdW epitaxially grown MoO_3_ generates an anisotropic strain in graphene (*13*, *14*, *38*, *39*).

We also measured the angle-resolved electrical transport in the 1L-MoO_3_/Gr device. The inset of Fig. 4D shows a device in which multiple electrodes of different angles are deposited around the 1L-MoO_3_ island. Figure 4D shows the transfer curves (*I*_DS_-*V*_G_) measured with different electrodes at specific angles, indicating a highly p-doped MoO_3_/Gr, as observed in the Raman measurement. In addition, the current levels and slopes of the transfer curves strongly depend on the measured orientation. Figure 4 (E to G) shows the p-doping concentration at *V*_G_ = 0 V, conductance (*G*), and field-effect mobility (μ_FE_) of MoO_3_/Gr in polar coordinates. The p-doping concentration was calculated using the equation $p=\frac{\varepsilon_{0}\varepsilon_{r}V_{\mathrm{CNP}}}{e\;t_{\mathrm{ox}}}$, where ε_0_, ε_r_, *V*_CNP_, *e*, and *t*_ox_ are the vacuum permittivity, relative permittivity of SiO_2_, charge neutrality point of the MoO_3_/Gr device, elemental charge, and thickness of SiO_2_, respectively. The doping level showed no orientation dependence because the doping of graphene was generated by the charge transfer between MoO_3_ and graphene (Fig. 4E). In contrast, both conductance and μ_FE_ exhibit anisotropy with 180° periodicity, as shown in Fig. 4 (F and G), respectively. Anisotropy conductance ratio (*G*_max_/*G*_min_) was 1.43 at *V*_G_ = 0 V, which is comparable to that of a representative in-plane anisotropic 2D material, black phosphorus (~1.5) (*45*–*47*). Similarly, μ_FE_ of MoO_3_/Gr showed a large anisotropy ratio (μ_FE,max_/μ_FE,min_) of 1.77, maintaining a high mobility of 8155 cm^2^ V^−1^ s^−1^ at *p* = 1 × 10^12^ cm^−2^. This result shows that in-plane electrical anisotropy can be achieved by epitaxially grown MoO_3_ even in symmetrical graphene with isotropic Dirac cones at *K* and *K′* points of the Brillouin zone (BZ) (*48*, *49*). The insulating properties of MoO_3_ indicate no electrical contribution to the conductivity of MoO_3_/Gr (fig. S4). Note that the vdW epitaxially grown MoO_3_ led to a high p-doping concentration of graphene without substantial degradation in the carrier mobility. As a result, MoO_3_/Gr shows low sheet resistance of ~133 ohms per square (figs. S5 and S6). Although the contact resistance of the MoO_3_/Gr is reduced by doping compared to graphene device, influence of contacts on the conductivity of MoO_3_/Gr was excluded because of small values (fig. S7).

### Anisotropic distortion of graphene band structure by MoO_3_ epilayer

To clarify the origin of the unconventional conductance anisotropy of MoO_3_/Gr, we performed first-principles calculations based on density functional theory (DFT). Anisotropic periodic potentials on graphene cause anisotropic deformation of its band structure (*50*, *51*). Accordingly, we expected that the epitaxially grown MoO_3_ layers would create anisotropic periodic potentials in graphene in close contact. The formation of a rectangular superlattice and uniaxial compressive strain, which was experimentally observed in MoO_3_/Gr, can induce atomic-scale corrugation in monolayer graphene. Therefore, the MoO_3_ epilayers can exert anisotropic periodic potentials on corrugated graphene, leading to an anisotropic deformation of the band structure in graphene.

**Fig. 5.**
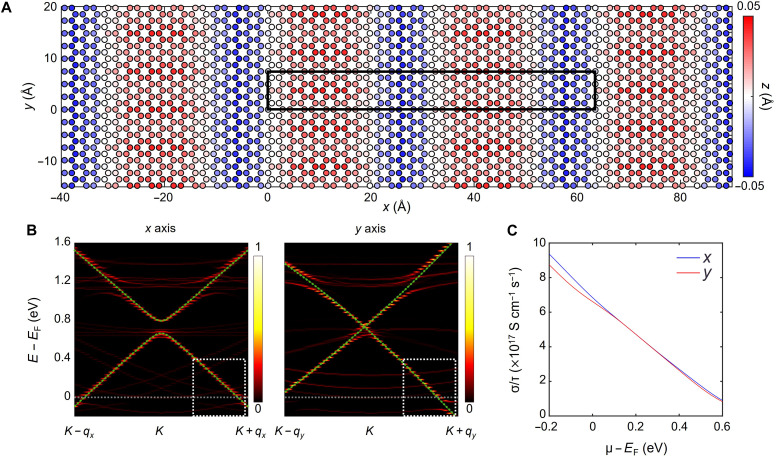
Anisotropic band distortion in MoO_3_/Gr. (**A**) Out-of-plane deformation of graphene for optimized MoO_3_/Gr heterostructure. Blue (red) color represents the displacement of carbon atoms below (above) the center of mass of the graphene layer. (**B**) Unfolded energy bands of the MoO_3_/Gr heterostructure, projected onto the graphene layer, along the *x* axis (left) and *y* axis (right) in the vicinity of the *K* point. The gray dashed lines represent the Fermi level of MoO_3_/Gr. The green dashed lines indicate the band structure of isolated graphene with the same compressive strain as that in the heterostructure, 1.2% along the *ac* direction and 0.2% along the *zz* direction. The color scale indicates the *k*-dependent spectral weight for the primitive BZ of the graphene layer. (**C**) Calculated in-plane electrical conductivities using the unfolded spectral functions projected on graphene at 300 K along the *x* and *y* axes.

To verify our hypothesis, we constructed a MoO_3_/Gr heterostructure by considering the strain induced by thermal expansion. The optimized lattice constants were 2.47 Å for monolayer graphene, and *a =* 3.96 Å and *c* = 3.70 Å for MoO_3_ monolayer. As observed in the TEM images in Fig. 2, the *a* and *c* axes of MoO_3_ are aligned with the *ac* and *zz* directions of graphene, respectively. A MoO_3_/Gr heterostructure with supercells of MoO_3_ (16 × *a* + 2 × *c*) and graphene (15 × *ac* + 3 × *zz*) was constructed by minimizing the mismatch between the lattice parameters of MoO_3_ and graphene and by applying orientation dependent TECs for MoO_3_ and graphene (*14*, *39*). In the constructed MoO_3_/Gr heterostructure, the graphene had anisotropic compressive strains of 1.2 and 0.2% along the *ac* and *zz* directions under the constraints of the MoO_3_ lattice, respectively. Figure 5A shows the out-of-plane deformation of graphene in contact with epitaxially grown 1L-MoO_3_. Within the superlattice (indicated by a black box), the MoO_3_-deposited graphene was corrugated with a maximum out-of-plane displacement of 0.09 Å. The carbon atoms that were aligned (misaligned) with the terminal oxygen atoms of MoO_3_ were relocated below (above) the mass center of the graphene layer. The upward and downward displacements of the carbon atoms in graphene are indicated in red and blue, respectively. Because of the atomic arrangement, graphene exhibited a 1D-like corrugation parallel to the *zz* direction, and the periodicity of the corrugation corresponded to the length of the superlattice.

The directional corrugation of graphene results in periodic potentials on graphene depending on the distance between MoO_3_ and graphene. In the supercell geometry, the electronic energy bands fold into small BZ of the cell so that the modifications of Dirac energy bands of graphene are hardly noticeable. To avoid this, we projected or unfolded the dense energy bands in the supercell BZ into the original BZ of graphene (*52*–*54*). Figure 5B shows the unfolded energy bands of the optimized MoO_3_/graphene heterostructure (fire contour) along two directions of *x* (*ac*) and *y* (*zz*), shown in Fig. 5A, projected onto the graphene layer. The Fermi level (*E*_F_; gray dashed lines) is downshifted by ~0.7 eV below the Dirac point of graphene, which supports our experimental observation of high p-doping concentration of graphene by the deposition of MoO_3_. We also compared the band structures of the isolated graphene layer (green dashed lines), which had the same compressive strain as the MoO_3_/graphene heterostructure. We noted that the apparent gapful (gapless) Dirac point along *q_x_* (*q_y_*) direction in Fig. 5B is caused by strain induced shift of Dirac point along *q_y_* direction. The effective band structure along the *y* axis on the right side of Fig. 5B is strongly altered at certain energy ranges, while that along the *x* axis on the left side of Fig. 5B is similar to that of isolated graphene (white dashed boxes). Around the Fermi level, band splitting is noticeable, indicating strong hybridization with the MoO_3_ layer. It is estimated that the directional electronic difference shown in Fig. 5B induces anisotropy in the electrical transport properties of the MoO_3_/graphene heterostructure.

On the basis of these band structures, the conductivities (σ) along the *x* and *y* axes could be estimated using the Boltzmann transport equation under a simple constant relaxation time (τ) approximation (*55*). Considering the crystal symmetry of the current system, the resulting conductivity along α(=x,y) direction (σ_α_) is given by

$$\sigma_{\alpha }(\mu ,T)=\frac{e^{2}\tau }{8\pi^{3}}\int \int \sum_{n}{\left[v_{n,k}^{\alpha }\right]}^{2}\delta (\varepsilon -\varepsilon_{n,k})\times \left[-\frac{\partial f(\varepsilon ,\mu ,T)}{\partial \varepsilon }\right]dkd\varepsilon$$ (1)

where **v**^α^_*n*,**k**_, ε_*n*,**k**_, τ, and *f* are the α-directional component of **k**-dependent group velocity, energy of the *n*th band, relaxation time (considered a constant for simplicity), and Fermi distribution function as a function of the energy (ε), chemical potential (μ), and temperature (*T*), respectively. As shown in Fig. 5C, the electrical conductivity along the *y* axis is lower than that along the *x* axis around the Fermi level, consistent with our experimental results. Although the simulation results partly support observed anisotropic conductance originated from structural disparities for two orthogonal directions, the obtained anisotropy is still smaller than the measurement. We may consider further detailed characteristics reflecting actual experiment situations, such as larger supercell geometries and different scattering times along the direction, to improve the simulation results later.

## DISCUSSION

In conclusion, we systemically investigated the interlayer interaction between MoO_3_ epilayers and monolayer graphene growth templates in the symmetry-mismatched epitaxy. Our results demonstrated that the extreme modulation of doping and strain in graphene was generated by the strong interlayer interaction with the MoO_3_ epilayers. First, the hole concentration in graphene could be markedly increased by the deposition of a single layer of MoO_3_. Our results were comparable with those obtained using other similar doping methods (deposition of a charge-transfer layer on graphene) (*56*, *57*). In addition, by modulating the thickness of the MoO_3_ epilayers, the strain exerted on graphene could be controlled, preserving a high hole concentration. Furthermore, the directional deformation of the graphene band structure leads to anisotropy in the electrical conductance of symmetric graphene. Overall, our work shows that the strong interlayer interaction between vdW epitaxially grown 2D oxides and 2D materials can be used as an approach for symmetry engineering of 2D materials, while preserving their outstanding electrical properties. Our findings have promising applications in optoelectronic devices that require optical and electrical anisotropy.

## MATERIALS AND METHODS

### Sample preparation

A MoO_3_/graphene heterostructure was prepared using our previously reported methods (*23*). As the Mo source, a 100-nm-thick Mo film was deposited on a SiO_2_ (285 nm)/Si substrate using an e-beam evaporator or a DC magnetron sputter. The quality and morphology of MoO_3_ were irrelevant to the metal deposition method. As the target substrate, graphene was mechanically exfoliated on another SiO_2_/Si substrate. Only monolayer graphene flakes were selected and used as target templates after their thicknesses were identified using Raman spectroscopy. To synthesize MoO_3_ on monolayer graphene, the Mo film was placed on a preheated heater (525°C), and, shortly after, the target substrate was located 0.5 mm above the Mo film upside down. The Mo film was oxidized and sublimated into MoO*_x_* and condensed on the graphene-exfoliated substrate because of the temperature difference between the source and target substrates. After 10 min, the target substrate was immediately removed from the heater. The thickness and coverage of MoO_3_ could be roughly controlled by varying the deposition time.

### Raman spectroscopy

Raman spectra were acquired using a Raman spectroscope (Renishaw Raman, inVia Reflex Confocal Raman Microscope, 532 nm, 600 gratings). Angle-resolved polarized Raman measurements were performed using a home-built confocal micro-Raman system with excitation sources of the 2.33 eV (532 nm) line from a diode-pumped solid-state laser. A 50× objective lens (0.8 numerical aperture) was used to focus the laser beam onto the sample and collect the scattered light (backscattering geometry). The Raman signal was dispersed using a Jobin-Yvon HORIBA iHR550 spectrometer (2400 grooves mm^−1^) and detected by a liquid nitrogen–cooled back-illuminated charge-coupled device detector. The laser power was kept below 0.1 mW. The polarizer was fixed at constant angle (θ_in_), and the analyzer angle (θ_out_) was rotated to select specific polarization of the scattered light. An achromatic half-wave plate was placed in front of the spectrometer to keep the polarization direction of the signal entering the spectrometer constant with respect to the groove direction of the grating.

### Transmission electron microscopy

MoO_3_/Gr was transferred to a holey carbon Au TEM grid using a poly(methyl methacrylate) (PMMA)–based transfer method. PMMA was spin-coated on the SiO_2_/Si substrate where MoO_3_/Gr was located and immersed in a 2 weight % KOH solution after scouring the edges of the substrate. The PMMA/MoO_3_/Gr film floated on the solution because SiO_2_ was etched by KOH. The film was rinsed with deionized water several times and transferred onto a holey carbon Au TEM grid, and PMMA was removed by placing the TEM grid in acetone overnight. High-resolution TEM images were captured using a Cs-TEM (JEOL JEM-ARM 200F Cs-TEM).

### Atomic force microscopy

AFM images were measured using NX-10 (Park Systems). Both contact and noncontact modes were performed considering the status of the samples and environment.

### Device fabrication and electrical measurements

e-beam lithography was performed to define the patterns of the source and drain electrodes surrounding MoO_3_/Gr. Subsequently, Cr/Pd/Au (2 nm/30 nm/40 nm) was deposited on MoO_3_/Gr using an e-beam evaporator. The electrical measurements of the devices were performed using a parameter analyzer (Keithley 2400) under ambient conditions. The two-probe field-effect mobility (μ_FE_) of MoO_3_/Gr was calculated using the following equation

$$\mu_{\mathrm{FE}}=g_{m}\frac{L}{WV_{\mathrm{DS}}C}$$ (2)

where *c* is the unit back-gate capacitance of 285-nm SiO_2_, *g*_m_ = *dI*_DS_/*dV*_G_ is the transconductance, *V*_DS_ is the drain voltage, and *L* and *W* are the channel length and width, respectively. The channel width is defined as the width of the metal electrodes. The transconductance was obtained by linearly fitting the transfer curve.

### DFT calculations

To investigate the electronic and transport properties of the MoO_3_/graphene heterostructure, we performed first-principles calculations based on DFT (*58*, *59*) using the VASP code (*60*, *61*). Projector-augmented wave potentials (*62*, *63*) were used to describe the valence electrons. The cutoff energy for the plane wave basis was set to 450 eV, and atomic relaxation was performed until the Hellmann-Feynman force acting on every atom decreased below 0.01 eV Å^−1^. Dipole correction was included for a more precise calculation. For the exchange-correlation function, the rev-vdW-DF2 method (*64*) was adopted to consider vdW interactions. The BZ was sampled using a 2 × 8 × 1 *k*-grid for the rectangular supercell of MoO_3_/graphene. To avoid spurious interlayer interaction along the out-of-plane direction, a vacuum region of 15 Å was introduced.

## Supplementary Materials

### This PDF file includes:

Supplementary Text Figs. S1 to S7 References
