# Supplementary material for: In-plane anisotropy of graphene by strong interlayer interactions with van der Waals epitaxially grown MoO3
Source: Sci Adv. 2023 Jun 7;9(23):eadg6696. doi: 10.1126/sciadv.adg6696 (PMC10246909; doi:10.1126/sciadv.adg6696)
Supplement: Supplementary file 1 — Supplementary Text Figs. S1 to S7 References [file sciadv.adg6696_sm.pdf]

Science Advances
AAAS

Supplementary Materials for

In-plane anisotropy of graphene by strong interlayer interactions with van der Waals epitaxially grown MoO_3

Hangyel Kim *et al.*

Corresponding author: Gwan-Hyoung Lee, gwanlee@snu.ac.kr

Sci. Adv. **9**, eadg6696 (2023)
DOI: 10.1126/sciadv.adg6696

This PDF file includes:

Supplementary Text
Figs. S1 to S7
References

Supplementary Text

1. Thickness estimation of MoO_3 fully covering graphene flake

The thickness of MoO_3 fully covering graphene flakes was estimated by measuring the height difference between the MoO_3/Gr heterostructure and SiO_2/Si substrate. As shown in Fig. S1, the height difference was measured as 1.78 nm, which corresponds to the sum of the thickness of bilayer MoO_3 (~1.4 nm) and monolayer graphene (0.33 nm).

2. Modified correlation plot for quantification of strain and hole exerted on graphene

The correlation plot for quantifying strain and hole in graphene was modified and extended based on our previous work *(32)*.

First, the origin of the plot was adjusted based on the linear dispersion of 2D peak position (ω_{2D}) as a function of the laser excitation energy (~100 cm^{-1} eV^{-1}) *(26, 27)*. The origin of the correlation plot was relocated from (ω_G^o, ω_{2D}^o)=(1581.6 cm^{-1}, 2676.9 cm^{-1}) to (1581.6 cm^{-1}, 2668.9 cm^{-1}), to account for the laser excitation energies used in reference *(32)* (514 nm=2.41 eV) and this study (532 nm=2.33 eV).

Second, we extrapolated the relation between p and ω_G for ω_G > 1602 cm^{-1}, which was not shown in our previous result *(32)*. We established the relation between p and ω_G using the following-equations:

$$E_F = \hbar\, v_F \sqrt{\pi p} \qquad \text{Eq. 1 } (65)$$

$$\Delta\omega_G = \alpha\, \Delta E_F \qquad \text{Eq. 2 } (66)$$

where E_F is fermi level of graphene, v_F is Fermi velocity of graphene, $\hbar$ is reduced Plank constant, and α is an empirical value. By combining these two equations, we can obtain quadratic relation between p and ω_G:

$$\Delta p = \frac{(\Delta\omega_G)^2}{\pi(\alpha\hbar v_F)^2}$$

$$p = \frac{(\omega'_G - \omega_G^o)^2}{\pi(\alpha\hbar v_F)^2} \qquad \text{Eq. 3}$$

where ω'_G is the position of G peak projected onto the hole axis parallel to the strain axis. To calculate p, appropriate ω_G^o and α should be determined. Among several references reporting the relation between doping and the change of G peak position *(65-68)*, we obtained ω_G^o and α from the results of the reference *(66)*, which were adopted in our previous work to plot p-axis (Fig. S2) *(32)*. Except for the small hole concentration region (ω_G<1584.5 cm^{-1}), the experimental results of reference *(32)* can be precisely fitted to equation 3, and we obtained ω_G^o=1570.67 cm^{-1} and α=61.7 cm^{-1} eV^{-1}. Based on these parameters, we calculated the hole concentrations of as-exfoliated graphene, uncovered graphene, and MoO_3/Gr in the main text.

3. Deconvolution of G peak in angle-resolved polarized Raman spectroscopy

As shown in Fig. 4B, the G peak appears to oscillate as a function of the analyzer angle θ_{out}. The oscillation results from the separation of G peak into G^- and G^+ and alternation of their intensities ($I(G^-)$ and $I(G^+)$) depending on the analyzer angle under uniaxial strain *(42-44)*. However, due to the small magnitude of the uniaxial strain, the two peaks are not fully separated. To define G^- and G^+, we first fitted the G peak with a single Lorentzian function and extracted ω_G for every θ_{out} (boxes) as shown in Fig. S3. The ω_G values match well with a sinusoidal function:

$$\omega_G = A \sin\left[\frac{\pi}{90}(\theta_{out} - \alpha)\right] + B \text{ (dashed line).}$$

We defined the maximum and minimum ω_G values as $\omega_{G^-}=B+A$ (red line) and $\omega_{G^+}=B-A$ (blue line), respectively. Note that ω_{G^+} have a smaller Raman shift than that of ω_{G^-}, because their analogy is adopted from that of nanotubes and tensioned graphene *(42-44)*.

Next, we fitted the G peak with double Lorentzian functions, with the fixed at ω_{G^-} and ω_{G^+} as shown in Fig. 4a in the main text. The obtained $I(G^-)$ and $I(G^+)$ values coincide with the references *(42-44)*, which demonstrates that uniaxial strain is exerted on MoO_3/Gr.

The magnitude of the uniaxial strain was calculated by difference in Grüneisen parameters of ω_{G^-} and ω_{G^+} $\left(\frac{\partial\,[\omega_{G^-} - \omega_{G^+}]}{\partial \varepsilon}\right)$. According to the reference *(44)*, the difference in Grüneisen parameters of ω_{G^-} and ω_{G^+} of graphene under uniaxial compression ranges from 16.8 to 23 $cm^{-1}\,\%^{-1}$. The separation of the peaks ($[\omega_{G^-}-\omega_{G^+}]$) in our sample is 1.46 cm^{-1}, thus it can be assumed that uniaxial compressive strain in our sample is around 0.06 to 0.09%.

4. Comparison of electrical properties of MoO_3 with MoO_3/Gr

To verify that the electrical anisotropy in our MoO_3/Gr samples did not originate from the anisotropy of MoO_3 itself, we measured the electrical properties of a MoO_3 field effect transistor (FET) device. We delaminated MoO_3 from MoO_3/Gr and transferred it to a bare SiO_2/Si substrate by dry transfer method with poly(bisphenol A carbonate, Sigma Aldrich) (Fig. S4A) *(69)*. The thickness of the transferred MoO_3 island (red square in Fig. S4A) was measured to be ~1.4 nm by AFM. Next, Cr/Pd/Au=2 nm/30 nm/40 nm electrodes, identical the electrodes used for MoO_3/Gr samples in the main text, were deposited on the MoO_3 island (Fig. S4B). The transfer characteristic of the MoO_3 FET device was measured under identical drain voltage (V_{DS}=4 mV) with the MoO_3/Gr devices (Fig. S4C). The MoO_3 FET showed highly insulating properties (<1 pA), indicating that the electrical conduction and anisotropy of MoO_3 can be neglected in MoO_3/Gr samples.

5. Sheet and contact resistances of MoO_3/Gr

We used transfer length method (TLM) to investigate sheet resistance (R_s) and contact resistance (R_c) of MoO_3/Gr. To investigate effect of MoO_3, we fabricated one graphene device which was annealed but without MoO_3 deposition (Fig. S5A) and two 1L-MoO_3/Gr devices (Fig. S5B and S5C).

Figure S6 shows R_s of the devices as a function of V_G. The MoO_3/Gr devices #1 and #2 displayed sheet resistances of 133 and 128 ohms/sq, respectively, at V_G=0V. This is considerably lower than that of the graphene device (241 ohms/sq).

On the other hand, as shown in Fig. S7, the graphene device shows R_c of 467 ohms·µm, whereas the contact resistances of the 1L-MoO_3/Gr devices were 268 and 350 ohms·µm, respectively, at V_G=0V. This suggests that deposition of MoO_3 reduces the contact resistance through contact area doping.

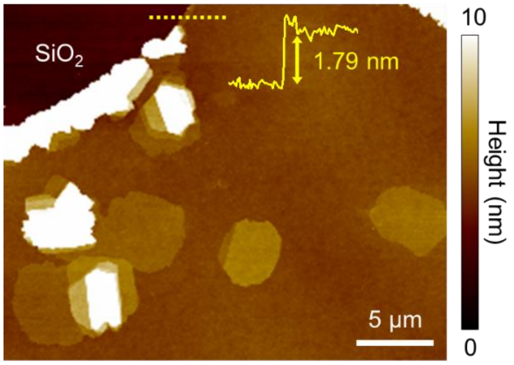

Fig. S1.

AFM topographic image of MoO_3/Gr. The thickness profile from the yellow-dashed line shows that bilayer MoO_3 with a thickness of 1.4 nm fully covers the monolayer graphene with a thickness of 0.33 nm.

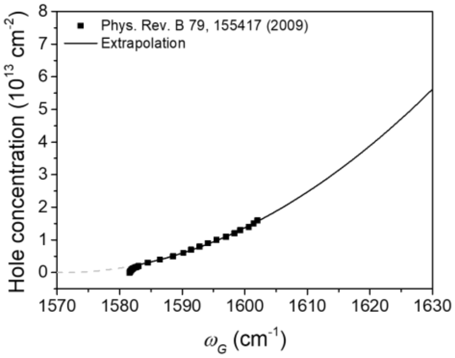

Fig. S2.

Extrapolation of hole axis of the correlation plot for high hole concentration region (ω_G>1602 cm^{-1}). The relation between ω_G and hole concentration was extrapolated based on their quadratic relation. The experimental results from reference *(66)* are represented by the boxes, while the solid line represents the extrapolated relation.

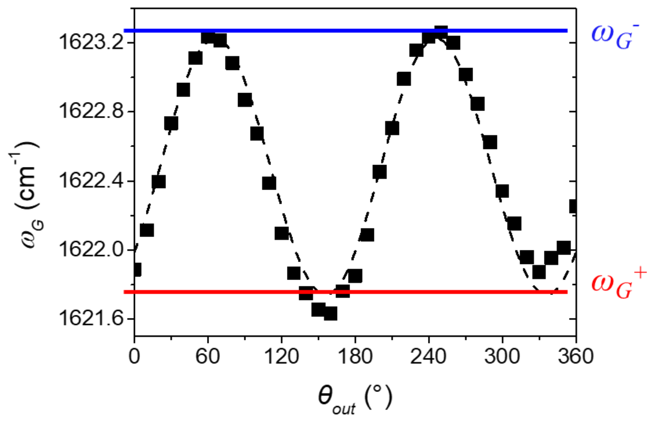

Fig. S3.

Deconvolution of G peak into G^+ and G^-. The position of G peak (ω_G) was fitted with the sinusoidal curve as a function of analyzer angle (θ_{out}) $\{\omega_G = A \sin\left[\frac{\pi}{90}(\theta_{out}-\alpha)\right] + B, A > 0\}$. The positions of split peaks (ω_{G^+} and ω_{G^-}) were defined as B-A and B+A, respectively.

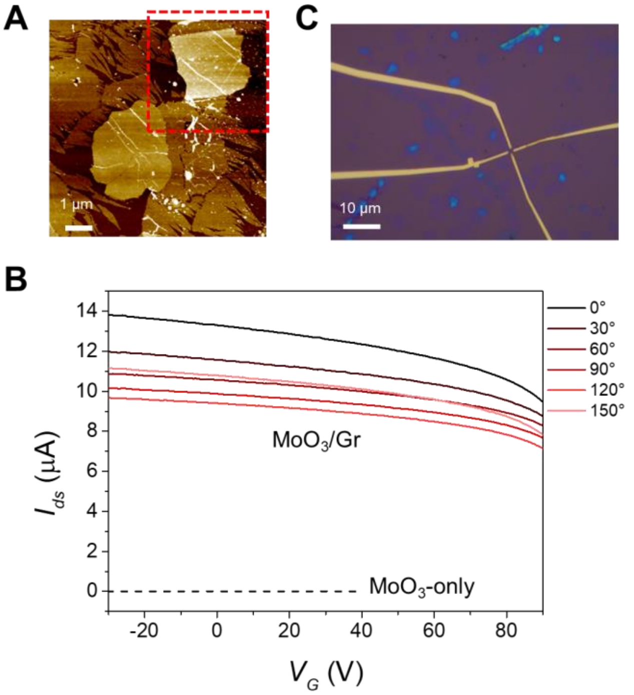

Fig. S4.

Transfer characteristics of MoO_3-only device. (A), AFM image of MoO_3 delaminated from MoO_3/graphene and transferred onto SiO_2/Si substrate. **(B),** Optical image of the MoO_3-only device with metal electrodes deposited on a 2L-MoO_3 island (marked with red square in Fig. S4A). **(C),** Transfer curves of MoO_3/Gr device for several orientations (solid lines) and MoO_3-only device (dashed line).

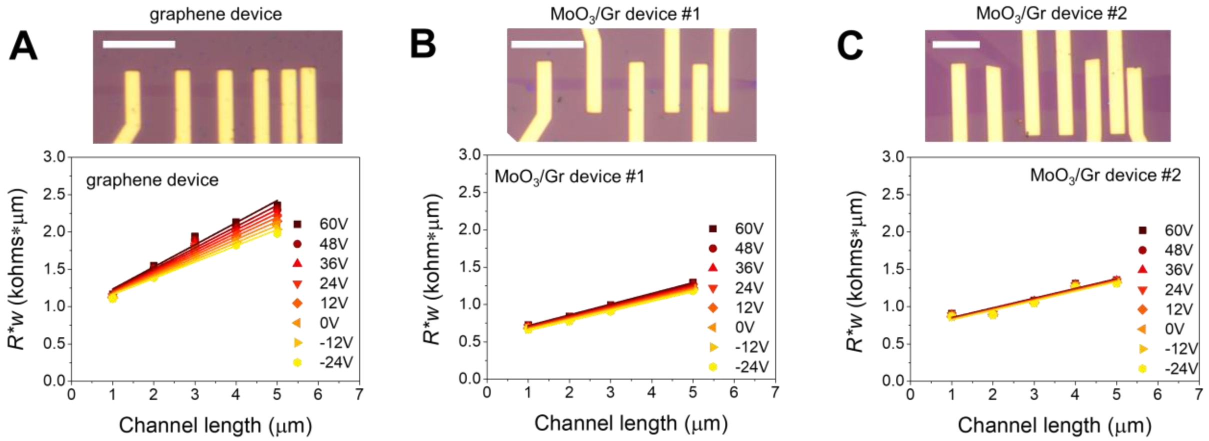

Fig. S5.

Optical microscope images and total resistance as a function of channel width of the TLM devices. (A), graphene device, (B), 1L-MoO_3/Gr device #1, and (C), 1L-MoO_3/Gr device #2. The scale bars are 10 μm.

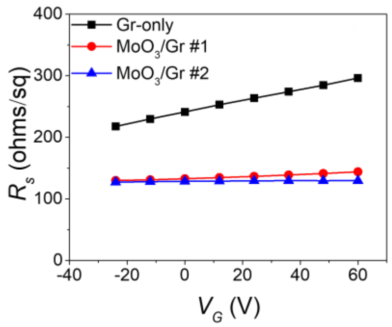

Fig. S6.
Sheet resistances of graphene device, 1L-MoO_3/Gr device #1, and 1L-MoO_3/Gr device #2, measured by TLM method.

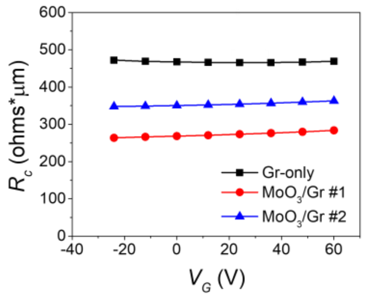

Fig. S7.
Contact resistances of graphene device, 1L-MoO_3/Gr device #1, and 1L-MoO_3/Gr device #2 measured by TLM method.

REFERENCES AND NOTES

1. L. A. Walsh, C. L. Hinkle, Van der Waals epitaxy: 2D materials and topological insulators. *Appl. Mater. Today* **9**, 504–515 (2017).

2. H. Kum, D. Lee, W. Kong, H. Kim, Y. Park, Y. Kim, Y. Baek, S.-H. Bae, K. Lee, J. Kim, Epitaxial growth and layer-transfer techniques for heterogeneous integration of materials for electronic and photonic devices. *Nat. Electron.* **2**, 439–450 (2019).

3. A. K. Geim, I. V. Grigorieva, Van der Waals heterostructures. *Nature* **499**, 419–425 (2013).

4. A. Koma, Van der Waals epitaxy—A new epitaxial growth method for a highly lattice-mismatched system. *Thin Solid Films* **216**, 72–76 (1992).

5. K. Ueno, K. Saiki, T. Shimada, A. Koma, Epitaxial growth of transition metal dichalcogenides on cleaved faces of mica. *J. Vac. Sci. Technol. A* **8**, 68–72 (1990).

6. Z. Lin, A. Yin, J. Mao, Y. Xia, N. Kempf, Q. He, Y. Wang, C.-Y. Chen, Y. Zhang, V. Ozolins, Scalable solution-phase epitaxial growth of symmetry-mismatched heterostructures on two-dimensional crystal soft template. *Sci. Adv.* **2**, e1600993 (2016).

7. G. Tang, P. You, Q. Tai, A. Yang, J. Cao, F. Zheng, Z. Zhou, J. Zhao, P. K. L. Chan, F. Yan, Solution-phase epitaxial growth of perovskite films on 2D material flakes for high-performance solar cells. *Adv. Mater.* **31**, e1807689 (2019).

8. Z. Wu, Y. Lyu, Y. Zhang, R. Ding, B. Zheng, Z. Yang, S. P. Lau, X. H. Chen, J. Hao, Large-scale growth of few-layer two-dimensional black phosphorus. *Nat. Mater.* **20**, 1203–1209 (2021).

9. K. Hermann, Periodic overlayers and moiré patterns: Theoretical studies of geometric properties. *J. Phys. Condens. Matter* **24**, 314210 (2012).

10. K. Tang, W. Qi, Moiré-pattern-tuned electronic structures of van der Waals heterostructures. *Adv. Funct. Mater.* **30**, 2002672 (2020).

11. Y. Liu, J. N. B. Rodrigues, Y. Z. Luo, L. Li, A. Carvalho, M. Yang, E. Laksono, J. Lu, Y. Bao, H. Xu, S. J. R. Tan, Z. Qiu, C. H. Sow, Y. P. Feng, A. H. Castro Neto, S. Adam, J. Lu, K. P. Loh, Tailoring sample-wide pseudo-magnetic fields on a graphene–black phosphorus heterostructure. *Nat. Nanotechnol.* **13**, 828–834 (2018).

12. T. Akamatsu, T. Ideue, L. Zhou, Y. Dong, S. Kitamura, M. Yoshii, D. Yang, M. Onga, Y. Nakagawa, K. Watanabe, T. Taniguchi, J. Laurienzo, J. Huang, Z. Ye, T. Morimoto, H. Yuan, Y. Iwasa, A van der Waals interface that creates in-plane polarization and a spontaneous photovoltaic effect. *Science* **372**, 68–72 (2021).

13. G. H. Ahn, M. Amani, H. Rasool, D.-H. Lien, J. P. Mastandrea, J. W. Ager Iii, M. Dubey, D. C. Chrzan, A. M. Minor, A. Javey, Strain-engineered growth of two-dimensional materials. *Nat. Commun.* **8**, 608 (2017).

14. H. Negishi, S. Negishi, Y. Kuroiwa, N. Sato, S. Aoyagi, Anisotropic thermal expansion of layered MoO_3 crystals. *Phys. Rev. B* **69**, 064111 (2004).

15. S. Puebla, R. D'Agosta, G. Sanchez-Santolino, R. Frisenda, C. Munuera, A. Castellanos-Gomez, In-plane anisotropic optical and mechanical properties of two-dimensional MoO_3. *npj 2D Mater. Appl.* **5**, 37 (2021).

16. W.-B. Zhang, Q. Qu, K. Lai, High-mobility transport anisotropy in few-layer MoO_3 and its origin. *ACS Appl. Mater. Interfaces* **9**, 1702–1709 (2017).

17. B. Zheng, Z. Wang, Y. Chen, W. Zhang, X. Li, Centimeter-sized 2D α-MoO_3 single crystal: Growth, Raman anisotropy, and optoelectronic properties. *2D Mater.* **5**, 045011 (2018).

18. W. Ma, P. Alonso-González, S. Li, A. Y. Nikitin, J. Yuan, J. Martín-Sánchez, J. Taboada-Gutiérrez, I. Amenabar, P. Li, S. Vélez, In-plane anisotropic and ultra-low-loss polaritons in a natural van der Waals crystal. *Nature* **562**, 557–562 (2018).

19. L. Kihlborg, Least squares refinement of crystal structure of molybdenum trioxide. *Ark. Kemi* **21**, 357 (1963).

20. G. Andersson, A. Magneli, L. Sillén, M. Rottenberg, On the crystal structure of molybdenum trioxide. *Acta Chem. Scand.* **4**, 793–797 (1950).

21. A. J. Molina-Mendoza, J. L. Lado, J. O. Island, M. A. Niño, L. Aballe, M. Foerster, F. Y. Bruno, A. Lopez-Moreno, L. Vaquero-Garzon, H. S. Van Der Zant, Centimeter-scale synthesis of ultrathin layered MoO_3 by van der Waals epitaxy. *Chem. Mater.* **28**, 4042–4051 (2016).

22. H. Ding, K. G. Ray, V. Ozolins, M. Asta, Structural and vibrational properties of α-MoO_3 from van der Waals corrected density functional theory calculations. *Phys. Rev. B* **85**, 012104 (2012).

23. J. H. Kim, J. K. Dash, J. Kwon, C. Hyun, H. Kim, E. Ji, G.-H. Lee, Van der Waals epitaxial growth of single crystal α-MoO_3 layers on layered materials growth templates. *2D Mater.* **6**, 015016 (2018).

24. M. A. Py, P. E. Schmid, J. T. Vallin, Raman scattering and structural properties of MoO_3. *Il Nuovo Cimento B (1971-1996)* **38**, 271–279 (1977).

25. M. Dieterle, G. Weinberg, G. Mestl, Raman spectroscopy of molybdenum oxides. *Phys. Chem. Chem. Phys.* **4**, 812–821 (2002).

26. A. C. Ferrari, D. M. Basko, Raman spectroscopy as a versatile tool for studying the properties of graphene. *Nat. Nanotechnol.* **8**, 235–246 (2013).

27. L. M. Malard, M. A. Pimenta, G. Dresselhaus, M. S. Dresselhaus, Raman spectroscopy in graphene. *Phys. Rep.* **473**, 51–87 (2009).

28. J. H. Kim, C. Hyun, H. Kim, J. K. Dash, K. Ihm, G.-H. Lee, Thickness-insensitive properties of α-MoO_3 nanosheets by weak interlayer coupling. *Nano Lett.* **19**, 8868–8876 (2019).

29. J. C. Meyer, A. K. Geim, M. I. Katsnelson, K. S. Novoselov, T. J. Booth, S. Roth, The structure of suspended graphene sheets. *Nature* **446**, 60–63 (2007).

30. Q. Yu, L. A. Jauregui, W. Wu, R. Colby, J. Tian, Z. Su, H. Cao, Z. Liu, D. Pandey, D. Wei, T. F. Chung, P. Peng, N. P. Guisinger, E. A. Stach, J. Bao, S.-S. Pei, Y. P. Chen, Control and characterization

of individual grains and grain boundaries in graphene grown by chemical vapour deposition. *Nat. Mater.* **10**, 443–449 (2011).

31. E. Ji, M. J. Kim, J.-Y. Lee, D. Sung, N. Kim, J.-W. Park, S. Hong, G.-H. Lee, Substrate effect on doping and degradation of graphene. *Carbon* **184**, 651–658 (2021).

32. J. E. Lee, G. Ahn, J. Shim, Y. S. Lee, S. Ryu, Optical separation of mechanical strain from charge doping in graphene. *Nat. Commun.* **3**, 1024 (2012).

33. S. Ryu, L. Liu, S. Berciaud, Y.-J. Yu, H. Liu, P. Kim, G. W. Flynn, L. E. Brus, Atmospheric oxygen binding and hole doping in deformed graphene on a SiO_2 substrate. *Nano Lett.* **10**, 4944–4951 (2010).

34. J. Son, M. Choi, J. Hong, I. S. Yang, Raman study on the effects of annealing atmosphere of patterned graphene. *J. Raman Spectrosc.* **49**, 183–188 (2018).

35. Y. Guo, J. Robertson, Origin of the high work function and high conductivity of MoO_3. *Appl. Phys. Lett.* **105**, 222110 (2014).

36. A. L. F. Cauduro, R. Dos Reis, G. Chen, A. K. Schmid, H.-G. Rubahn, M. Madsen, Work function mapping of MoO_x thin-films for application in electronic devices. *Ultramicroscopy* **183**, 99–103 (2017).

37. D. A. Kowalczyk, M. Rogala, K. Szalowski, W. Kozlowski, I. Lutsyk, M. Piskorski, P. Krukowski, P. Dabrowski, D. Belić, M. Cichomski, Z. Klusek, P. J. Kowalczyk, Local electronic structure of stable monolayers of α-MoO_{3-x} grown on graphite substrate. *2D Mater.* **8**, 025005 (2020).

38. Y. Hu, X. Liu, S. Xu, W. Wei, G. Zeng, H. Yuan, Q. Gao, J. Guo, M. Chao, E. Liang, Improving the thermal expansion and capacitance properties of MoO_3 by introducing oxygen vacancies. *J. Phys. Chem. C* **125**, 10817–10823 (2021).

39. D. Yoon, Y.-W. Son, H. Cheong, Negative thermal expansion coefficient of graphene measured by Raman spectroscopy. *Nano Lett.* **11**, 3227–3231 (2011).

40. T. Liang, W. G. Sawyer, S. S. Perry, S. B. Sinnott, S. R. Phillpot, First-principles determination of static potential energy surfaces for atomic friction in MoS2 and MoO3. *Phys. Rev. B* **77**, 104105 (2008).

41. T. Korhonen, P. Koskinen, Peeling of multilayer graphene creates complex interlayer sliding patterns. *Phys. Rev. B* **92**, 115427 (2015).

42. M. Huang, H. Yan, C. Chen, D. Song, T. F. Heinz, J. Hone, Phonon softening and crystallographic orientation of strained graphene studied by Raman spectroscopy. *Proc. Natl. Acad. Sci. U.S.A.* **106**, 7304–7308 (2009).

43. T. M. G. Mohiuddin, A. Lombardo, R. R. Nair, A. Bonetti, G. Savini, R. Jalil, N. Bonini, D. M. Basko, C. Galiotis, N. Marzari, K. S. Novoselov, A. K. Geim, A. C. Ferrari, Uniaxial strain in graphene by Raman spectroscopy: ***G*** peak splitting, Grüneisen parameters, and sample orientation. *Phys. Rev. B* **79**, 205433 (2009).

44. O. Frank, G. Tsoukleri, J. Parthenios, K. Papagelis, I. Riaz, R. Jalil, K. S. Novoselov, C. Galiotis, Compression behavior of single-layer graphenes. *ACS Nano* **4**, 3131–3138 (2010).

45. A. Castellanos-Gomez, Black phosphorus: Narrow gap, wide applications. *J. Phys. Chem. Lett.* **6**, 4280–4291 (2015).

46. H. Liu, A. T. Neal, Z. Zhu, Z. Luo, X. Xu, D. Tománek, P. D. Ye, Phosphorene: An unexplored 2D semiconductor with a high hole mobility. *ACS Nano* **8**, 4033–4041 (2014).

47. F. Xia, H. Wang, Y. Jia, Rediscovering black phosphorus as an anisotropic layered material for optoelectronics and electronics. *Nat. Commun.* **5**, 4458 (2014).

48. P. R. Wallace, The band theory of graphite. *Phys. Rev.* **71**, 622 (1947).

49. A. H. C. Neto, F. Guinea, N. M. R. Peres, K. S. Novoselov, A. K. Geim, The electronic properties of graphene. *Rev. Mod. Phys.* **81**, 109 (2009).

50 C.-H. Park, L. Yang, Y.-W. Son, M. L. Cohen, S. G. Louie, Anisotropic behaviours of massless Dirac fermions in graphene under periodic potentials. *Nat. Phys.* **4**, 213–217 (2008).

51 Y. Li, S. Dietrich, C. Forsythe, T. Taniguchi, K. Watanabe, P. Moon, C. R. Dean, Anisotropic band flattening in graphene with one-dimensional superlattices. *Nat. Nanotechnol.* **16**, 525–530 (2021).

52 P. V. C. Medeiros, S. Stafström, J. Björk, Effects of extrinsic and intrinsic perturbations on the electronic structure of graphene: Retaining an effective primitive cell band structure by band unfolding. *Phys. Rev. B* **89**, 041407 (2014).

53. P. V. C. Medeiros, S. S. Tsirkin, S. Stafström, J. Björk, Unfolding spinor wave functions and expectation values of general operators: Introducing the unfolding-density operator. *Phys. Rev. B* **91**, 041116 (2015).

54. S. Kim, J. Ihm, H. J. Choi, Y.-W. Son, Origin of anomalous electronic structures of epitaxial graphene on silicon carbide. *Phys. Rev. Lett.* **100**, 176802 (2008).

55. G. K. H. Madsen, J. Carrete, M. J. Verstraete, BoltzTraP2, a program for interpolating band structures and calculating semi-classical transport coefficients. *Comput. Phys. Commun.* **231**, 140–145 (2018).

56. M. S. Choi, A. Nipane, B. S. Y. Kim, M. E. Ziffer, I. Datta, A. Borah, Y. Jung, B. Kim, D. Rhodes, A. Jindal, Z. A. Lamport, M. Lee, A. Zangiabadi, M. N. Nair, T. Taniguchi, K. Watanabe, I. Kymissis, A. N. Pasupathy, M. Lipson, X. Zhu, W. J. Yoo, J. Hone, J. T. Teherani, High carrier mobility in graphene doped using a monolayer of tungsten oxyselenide. *Nat. Electron.* **4**, 731–739 (2021).

57. S.-J. Kwon, T.-H. Han, T. Y. Ko, N. Li, Y. Kim, D. J. Kim, S.-H. Bae, Y. Yang, B. H. Hong, K. S. Kim, S. Ryu, T.-W. Lee, Extremely stable graphene electrodes doped with macromolecular acid. *Nat. Commun.* **9**, 2037 (2018).

58. P. Hohenberg, W. Kohn, Inhomogeneous electron gas. *Phys. Rev.* **136**, B864 (1964).

59. W. Kohn, L. J. Sham, Self-consistent equations including exchange and correlation effects. *Phys. Rev.* **140**, A1133 (1965).

60. G. Kresse, J. Furthmüller, Efficient iterative schemes for ab initio total-energy calculations using a plane-wave basis set. *Phys. Rev. B* **54**, 11169 (1996).

61. G. Kresse, J. Hafner, Ab initio molecular dynamics for liquid metals. *Phys. Rev. B* **47**, 558 (1993).

62. P. E. Blöchl, Projector augmented-wave method. *Phys. Rev. B* **50**, 17953 (1994).

63. G. J. Kresse, D. Joubert, From ultrasoft pseudopotentials to the projector augmented-wave method. *Phys. Rev. B* **59**, 1758–1775 (1999).

64. I. Hamada, Van der Waals density functional made accurate. *Phys. Rev. B* **89**, 121103 (2014).

65. C.-F. Chen, C.-H. Park, B. W. Boudouris, J. Horng, B. Geng, C. Girit, A. Zettl, M. F. Crommie, R. A. Segalman, S. G. Louie, F. Wang, Controlling inelastic light scattering quantum pathways in graphene. *Nature* **471**, 617–620 (2011).

66. A. Das, B. Chakraborty, S. Piscanec, S. Pisana, A. K. Sood, A. C. Ferrari, Phonon renormalization in doped bilayer graphene. *Phys. Rev. B* **79**, 155417 (2009).

67. A. Das, S. Pisana, B. Chakraborty, S. Piscanec, S. K. Saha, U. V. Waghmare, K. S. Novoselov, H. R. Krishnamurthy, A. K. Geim, A. C. Ferrari, A. K. Sood, Monitoring dopants by Raman scattering in an electrochemically top-gated graphene transistor. *Nat. Nanotechnol.* **3**, 210–215 (2008).

68. M. Lazzeri, F. Mauri, Nonadiabatic Kohn anomaly in a doped graphene monolayer. *Phys. Rev. Lett.* **97**, 266407 (2006).

69. J. Son, J. Kwon, S. Kim, Y. Lv, J. Yu, J.-Y. Lee, H. Ryu, K. Watanabe, T. Taniguchi, R. Garrido-Menacho, N. Mason, E. Ertekin, P. Y. Huang, G.-H. Lee, A. M. van der Zande, Atomically precise graphene etch stops for three dimensional integrated systems from two dimensional material heterostructures. *Nat. Commun.* **9**, 3988 (2018).
